# Save the Bladder: Continent Urinary Diversion as Best Practice for Patients With Refractory Neurogenic Lower Urinary Tract Dysfunction

**DOI:** 10.1002/nau.70196

**Published:** 2025-12-09

**Authors:** Brian W. Chao, Sean P. Elliott

**Affiliations:** ^1^ Department of Urology University of Minnesota Medical School Minneapolis Minnesota USA

**Keywords:** continent, intermittent urethral catheterization, neurogenic, urinary bladder, urinary diversion, urinary reservoir

## Abstract

**Introduction:**

Refractory neurogenic lower urinary tract dysfunction presents an obstinate clinical problem. For patients who fail nonsurgical measures, we advocate for bladder‐sparing, continent urinary diversion as the optimal approach to management.

**Methods:**

The arguments presented herein were informed by a review of the contemporary literature and our longitudinal institutional experience caring for patients with neurogenic lower urinary tract dysfunction.

**Results:**

Bladder‐sparing, continent urinary diversion comprises procedures such as bladder augmentation, creation of a continent catheterizable channel, and bladder neck surgeries such as reconstruction, closure, and placement of a compressive sling or artificial sphincter. Such procedures facilitate clean intermittent catheterization, improve continence, and support patients' independent bladder management. These benefits yield quantifiable gains in quality of life, as demonstrated through several contemporary investigations incorporating patient‐reported outcomes measures. Moreover, while continent and non‐continent diversions are both susceptible to long‐term complications, the former can be managed through both surgical and non‐surgical means.

**Conclusions:**

Continent urinary diversion prioritizes patients' autonomy and quality of life. It should be considered the primary surgical option for patients with refractory neurogenic lower urinary tract dysfunction.

## 1 Introduction

Patients with neurogenic lower urinary tract dysfunction (NLUTD) face myriad challenges. Common urologic complications include urinary tract infections (UTIs, occurring roughly 2.5 times per patient per year), bladder calculi (affecting an estimated 49%), hydronephrosis (47%), and vesicoureteral reflux (33%) [1, 2, 3]. Feared sequelae such as upper tract deterioration and end‐stage renal disease (8%–24%) may be hastened by recurrent pyelonephritis and impaired bladder compliance [4, 5]. For many patients, equally vexing are the comparatively mundane challenges of daily life: the cumbersome process of clean intermittent catheterization (CIC), episodes of incontinence, and reliance on caregivers.

When conservative measures such as anticholinergics and botulinum toxin fail, patients and providers may pivot to surgical options for NLUTD. Broadly speaking, these can be characterized as extirpative versus bladder‐sparing and continent versus non‐continent. Bladder‐sparing, continent diversions encompass procedures such as bladder augmentation, creation of a continent catheterizable channel, and bladder neck surgery (e.g., reconstruction, sling, and artificial sphincter) for continence. Through our experience caring for the NLUTD population, we have come to favor bladder‐sparing, continent diversion as the initial surgical option for appropriately selected patients. These procedures address the central challenges of NLUTD, prioritize patients' autonomy and quality of life (QOL), and avoid long‐term complications associated with non‐continent diversion.

### 1.1 Challenges of NLUTD

Guidelines consistently recommend CIC as the optimal management strategy for patients with NLUTD [6, 7, 8]. However, data from the National Spinal Cord Injury (SCI) Database suggest that this practice is rarely sustained, with only 20% of patients continuing long‐term CIC [9]. Other authors have replicated these findings; oft‐cited reasons for cessation include incontinence, dependence on caregivers, constraints on daily activity, and a perceived association between CIC and UTIs [10, 11, 12].

Continent diversion, in its many forms, offers practical solutions for these drawbacks and a means to support and prioritize long‐term CIC. Bladder augmentation increases capacity and reduces intravesical pressures, improving continence and mitigating recurrent UTIs [13]. Myers et al. (2019), drawing from a population‐based registry of people with SCI and NLUTD, found that CIC with bladder augmentation improved continence and urinary symptoms as compared to CIC alone or with botulinum toxin [14]. If neurogenic sphincter deficiency, urethral erosion, or other pathologies coexist, bladder neck sling, closure, or artificial urinary sphincter can similarly address leakage [15, 16, 17].

Continent catheterizable channels fill a complementary need. A well‐placed channel and stoma can ease the process of CIC, reducing caregiver dependence and time spent catheterizing [18]. This is particularly salient for women (with any neurologic lesion) and quadriplegic men, who are at elevated risk for CIC cessation due to anatomic constraints and impaired upper extremity function [19, 20].

### 1.2 Quality of Life

It is critical to recognize that, despite functional limitations, the health and life priorities of patients with NLUTD mirror our own [21]. Continent urinary diversion respects these priorities by maximizing patients' autonomy and QOL.

Multiple studies have demonstrated QOL improvements and high satisfaction rates following bladder augmentation and creation of continent catheterizable channels [18, 22, 23, 24]. These effects appear durable, with high QOL scores reported even at a median follow‐up of 22 years [25]. While QOL improvements appear primarily driven by decreased urinary incontinence, multiple studies cite newfound independence with CIC as another key contributor [26, 27]. Other benefits include improvements in self‐image, self‐esteem, and sexuality [27, 28, 29].

A notable limitation of the current literature is the lack of comparative data between continent and non‐continent diversions for NLUTD. In the radical cystectomy literature, recent data suggest a trend towards higher QOL with orthotopic neobladders as compared to ileal conduits, comprising improved physical function, social function, and global health status [30, 31, 32, 33]. However, it must be noted that compared to oncologic patients, those with NLUTD have highly disparate baseline QOL, caregiver needs, and treatment goals [34]. Direct extrapolation of these data is therefore imprudent.

### 1.3 Longevity

The components of continent diversions exist in a delicate equilibrium – even small insults can interfere with CIC, worsen continence, and complicate patients' bladder management. When complications do arise, however, most can be managed conservatively and with reasonable outcomes. Stomal stenosis can be temporized with L‐stents to permit continued CIC [35]. When pathologies such as stomal stenosis (13%–55%), false passages (2%–4%), and incontinence (4%–42%) warrant reoperation, revision can be accomplished with good success rates [36, 37, 38, 39]. In our institutional series, 76% of patients with difficult‐to‐catheterize continent channels were revised successfully, with 89% overall retaining channel function at last follow‐up [40]. Bladder calculi, a common complication (23%–52%) of bladder augmentation, can be managed effectively and with minimal morbidity via endoscopic and percutaneous lithotripsy [41, 42, 43, 44, 45]. Moreover, we routinely implement preventive strategies such as bladder irrigation and periodic surveillance cystoscopies, which further reduce the incidence of calculi [46, 47].

Non‐continent diversions are not immune to long‐term complications. One large (> 1000 patients), long‐term study of ileal and colon conduit diversions found an overall complication rate of 61%, comprising bowel complications (20%), renal complications (20%), infectious complications (17%), stomal complications (15%), and urolithiasis (15%) [48]. Parastomal hernias affect an estimated 4%–35%, with high recurrence rates (27%–50%) despite repair [49]. Stomal stenosis (2%–8%) and mid‐conduit stenosis (3%) are comparatively rare but generally warrant reoperation; if long‐segment conduit stenosis is encountered, this requires replacement with a new bowel segment [50, 51]. Ureteroenteric anastomotic strictures develop in 7%–14% and can have profound impact through recurrent infections, loss of renal function, and reliance on indwelling stents and nephrostomy tubes [52]. Concerningly, high rates of renal function deterioration have been noted after ileal conduit diversion in children, reflected in the procedure's abandonment by the late 20th century [53, 54].

Ultimately, both continent and non‐continent urinary diversions are susceptible to long‐term complications. A critical advantage of beginning with continent diversion is preserving the option of future conversion to an ileal conduit. The reverse (i.e., urinary “undiversion”) is fraught with technical difficulty and morbidity [55, 56, 57].

## 2 Conclusion

For patients with refractory NLUTD, surgery presents an opportunity to restore independence and improve QOL. While no surgery is without risk, continent urinary diversion addresses the physiologic challenges of NLUTD while prioritizing autonomy, flexibility, and longevity. Whenever feasible, it should be considered as the first‐line surgical option.

## Author Contributions

**Brian Chao:** writing – original draft, writing – reviewing and editing. **Sean P. Elliott:** writing – reviewing and editing, supervision.

## Disclosure

All authors have read and approved the submitted version of this manuscript and attest to the integrity of the work presented.

## Ethics Statement

No ethical approval was required for this study as it does not involve original research with human participants or identifiable data.

## Conflicts of Interest

Sean Elliott has the following disclosures: speaker and consultant for Boston Scientific, consultant and principal investigator of clinical trial for Urotronic. Brian Chao has no financial disclosures.

## Data Availability

Data sharing is not applicable to this article as no data sets were generated or analysed during the current study.
